# Nodule Worm Infection in Humans and Wild Primates in Uganda: Cryptic Species in a Newly Identified Region of Human Transmission

**DOI:** 10.1371/journal.pntd.0002641

**Published:** 2014-01-09

**Authors:** Ria R. Ghai, Colin A. Chapman, Patrick A. Omeja, T. Jonathan Davies, Tony L. Goldberg

**Affiliations:** 1 Department of Biology, McGill University, Montreal, Quebec, Canada; 2 Department of Anthropology and McGill School of Environment, Montreal, Quebec, Canada, and Wildlife Conservation Society, Bronx, New York, New York, United States of America; 3 Makerere University Biological Field Station, Fort Portal, Uganda; 4 Department of Pathobiological Sciences, School of Veterinary Medicine, University of Wisconsin-Madison, Madison, Wisconsin, United States of America; University of Melbourne, Australia

## Abstract

**Introduction:**

Soil-transmitted helminths (STHs) are a major health concern in tropical and sub-tropical countries. *Oesophagostomum* infection is considered endemic to West Africa but has also been identified in Uganda, East Africa, among primates (including humans). However, the taxonomy and ecology of *Oesophagostomum* in Uganda have not been studied, except for in chimpanzees (*Pan troglodytes*), which are infected by both *O. bifurcum* and *O. stephanostomum*.

**Methods and Findings:**

We studied *Oesophagostomum* in Uganda in a community of non-human primates that live in close proximity to humans. Prevalence estimates based on microscopy were lower than those based on polymerase chain reaction (PCR), indicating greater sensitivity of PCR. Prevalence varied among host species, with humans and red colobus (*Procolobus rufomitratus*) infected at lowest prevalence (25% and 41% by PCR, respectively), and chimpanzees, olive baboons (*Papio anubis*), and l'hoest monkeys (*Cercopithecus lhoesti*) infected at highest prevalence (100% by PCR in all three species). Phylogenetic regression showed that primates travelling further and in smaller groups are at greatest risk of infection. Molecular phylogenetic analyses revealed three cryptic clades of *Oesophagostomum* that were not distinguishable based on morphological characteristics of their eggs. Of these, the clade with the greatest host range had not previously been described genetically. This novel clade infects humans, as well as five other species of primates.

**Conclusions:**

Multiple cryptic forms of *Oesophagostomum* circulate in the people and primates of western Uganda, and parasite clades differ in host range and cross-species transmission potential. Our results expand knowledge about human *Oesophagostomum* infection beyond the West African countries of Togo and Ghana, where the parasite is a known public health concern. *Oesophagostomum* infection in humans may be common throughout Sub-Saharan Africa, and the transmission of this neglected STH among primates, including zoonotic transmission, may vary among host communities depending on their location and ecology.

## Author Summary

Nodule worms infect the gastrointestinal tracts of a number of mammalian species, including humans and other primates. This study sought to identify the species of nodule worms causing infections within and around an East African national park in Uganda where monkeys and apes co-occur and overlap with people. Some primates, particularly those traversing large distances in small groups, were most susceptible to nodule worm infection. Additionally, molecular analyses identified three separate groups of nodule worm that could not be distinguished based on microscopic examination of their eggs. One of these groups was found in humans as well as other primates and had not previously been genetically characterized. These results suggest that certain types of nodule worm may be restricted to particular hosts, while others may be transmitted among primates, including humans. Nodule worms are currently thought to be a human health concern only in some West African countries. This research suggests that nodule worms have a broader geographic impact in humans than previously appreciated.

## Introduction

Soil-transmitted helminths (STHs) are parasitic nematodes that cause infection *via* eggs and larvae, which are shed in feces and persist in the soils of tropical and sub-tropical countries [1]. STHs infect over one billion people worldwide [2] and may cause a combined disease burden as substantial as that caused by malaria or tuberculosis [3]. Nevertheless, these parasites are largely neglected in research, perhaps in part because the diseases they cause are suffered by the world's most impoverished populations [1]. Although roundworms (*Ascaris lumbricoides*), hookworms (*Necator americanus* and *Ancylostoma duodenale*) and whipworms (*Trichuris trichiura*) are of global importance, other “lesser” parasites are localized to specific regions [1]. This includes *Oesophagostomum* spp., a genus of nodule-causing worms with L3 larvae that are infective *via* ingestion after 4–7 days [4]–[7]. The human burden of *Oeosphagostostomum* infection is considered localized to West Africa, specifically the countries of Togo and Ghana [5], [8], [9].

A variety of mammals, including pigs, ruminants [10], [11], and non-human primates are frequently parasitized by *Oesophagostomum*. Infections in wild primates appear to be asymptomatic; clinical signs and mortality due to *Oesophagostomum* have only been recorded in captive settings [10], [12]. Eight species of *Oesophagostomum* have been recorded in wild primates, of which the three most common, *O. bifurcum*, *O. stephanostomum*, and *O. aculeatum*, are able to infect humans [5], [7], [11], [13]. Of these, *O. bifurcum* appears to be the only species to regularly parasitize humans, with human infections by other species considered incidental [5], [9]. In Togo and Ghana, the majority of human *Oesophagostomum* cases occur within endemic foci [5], [8] and affect 20% and 90% of the population, respectively, with prevalence highest in rural areas [12], [14], [15]. The only known species to cause infection within these countries is *O. bifurcum*, which also infects the region's non-human primates, including patas monkeys (*Erythrocebus patas*), mona monkeys (*Cercopithecus mona*), and olive baboons (*Papio anubis*) [4], [10], [16]. However, previous research has indicated that *O. bifurcum* is not commonly transmitted among primate species (including humans) because different parasite variants within the species are adapted to specific hosts [4], [16], [17].

In Uganda, a number of primate species harbor *Oesophagostomum*, as evidenced by microscopic detection of eggs in feces. These include members of the primate subfamilies Cercopithecinae and Colobinae, as well as chimpanzees (*Pan trogolodytes*) [18]–[20]. There have also been reports of oesophagostomiasis in human patients in Uganda, although no such reports, to our knowledge, have been published since the 1980s [5], [21], perhaps due to under-reporting or improvements in treatment. With the exception of chimpanzees, which are infected with both *O. bifurcum* and *O. stephanostomum* [22], the species of *Oesophagostomum* infecting Ugandan primates and humans remains unknown.

In this study, we examined *Oesophagostomum* within the primate community of Kibale National Park, Uganda, using a combination of microscopic and molecular methods. Species-specific identification of eggs by microscopy alone is difficult, because eggs are similar morphologically to other STHs, including hookworms, *Trichostrongylus* spp., and the “false hookworm” *Ternidens deminutus* [5], [9], [23]–[25]. In other studies, coproculture of L3 larvae or necropsy to isolate adult worms have been used to identify these parasites to species [25], [26]. Here, we used molecular methods to detect and sequence *Oesophagostomum* DNA directly from feces; such methods have proven informative for other similar studies [27], [28]. In addition, we used phylogenetic comparative methods to ascertain whether primate host traits explain variation in prevalence of *Oesophagostomum* infection among host species. Our sampling and analyses included nearby human populations to assess whether *Oesophagostomum* is a public health concern in the region, as well the parasite's local propensity for zoonotic transmission.

## Methods

### Ethics Statement

Prior to data collection, all protocols were reviewed and approved by the Uganda National Council for Science and Technology and the Uganda Wildlife Authority, as well as by the Institutional Review Board and the Animal Care and Use Committees of McGill University and the University of Wisconsin-Madison. Due to low literacy rates, oral informed consent was obtained from all adult subjects and a parent or guardian of all minor participants by trained local field assistants and documented by witnessed notation on IRB-approved enrollment forms.

### Study Site and Sample Collection

Kibale National Park (0°13′–0°41′N, 30°19′–30°32′E) is a 795 km^2^ semi-deciduous protected area in Western Uganda. Primate research has occurred in Kibale for over four decades, focusing on chimpanzees and red colobus monkeys (*Procolobus rufomitratus*) [29], [30]. As a result, a number of primate groups are habituated to human presence, and many individuals are recognizable based on a combination of physical attributes and collars affixed as part of a larger project on primate health and conservation [31].

**Figure 1:**
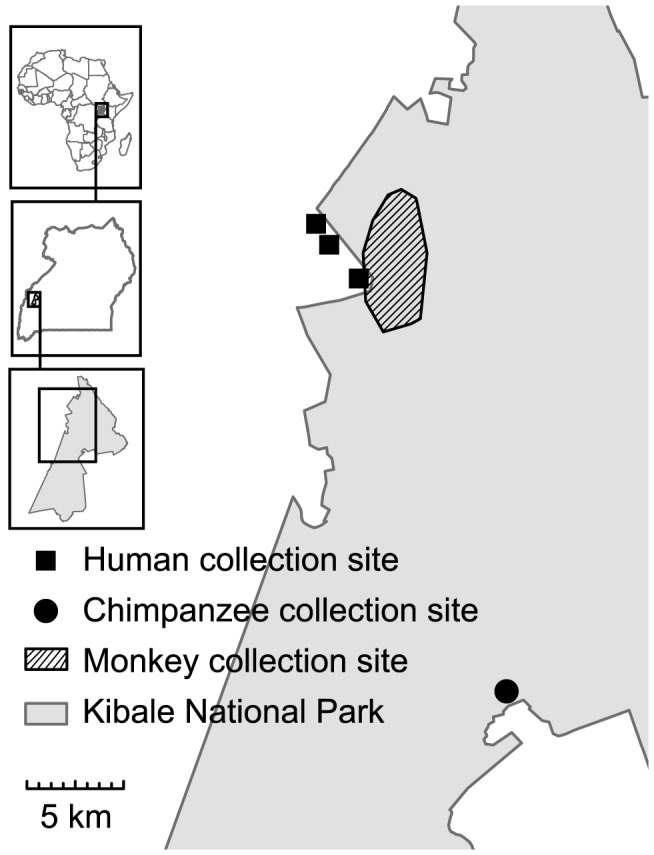
Map of sample collection sites in and around Kibale National Park, Uganda.

Samples from monkeys in the Kanyawara area of Kibale National Park were collected from red-tailed guenons (*Cercopithecus ascanius*), blue monkeys (*Cercopithecus mitis*), l'hoest monkeys (*Cercopithecus lhoesti*), grey-cheeked mangabeys (*Lophocebus albigena*), olive baboons (*Papio anubis*), red colobus, and black and white colobus (*Colobus guereza*) (Figure 1). Chimpanzee samples were collected from Kanyanchu, an area that has a habituated chimpanzee community as a result of tourism (Figure 1). All samples were collected non-invasively immediately after defecation and placed into sterile tubes. Date, location, species, age and sex category, and social group membership were recorded. Human samples were collected after the receipt of Institutional Review Board-approved informed consent following World Health Organisation protocols. Samples collection occurred in three villages: Ibura, Kanyansohera, and Kasojo, which are less than 5 km from the border of the park (Figure 1). Individuals between the ages of 2 and 70 were suitable participants of this study. Consenting participants were given instructions on how to collect the sample, which was then retrieved for processing within one day.

Samples were subjected to a modified ethyl acetate concentration method, recommended in the approved guidelines of the Clinical and Laboratory Standards Institute for the identification of intestinal-tract parasites [32], [33]. Concentration by sedimentation was performed in the field using one gram of undiluted feces without fixture in formalin, as formalin is a known inhibitor of the polymerase chain reaction [34]. All materials were sterilized prior to use, and care was exercised throughout the procedure to prevent contamination. Sediments were left uncapped for two hours after completion of the procedure to allow ethyl acetate that may inhibit polymerase chain reaction (PCR) to volatilize. Sediments were then suspended in 2 mL RNALater nucleotide stabilization solution (Sigma-Aldrich, St. Louis, MO, USA) and frozen at −20°C until shipment to North America.

### Microscopy

Thin smears from sedimented feces were used for microscopy [35]. All eggs of the genus *Oesophagostomum* were identified at 10× objective magnification on a Leica DM2500 light microscope. Data were recorded on size, shape, color and internal contents of eggs. Images were captured at 40× objective magnification of all specimens using an Infinity1 CMOS digital microscope camera and Infinity Camera v.6.2.0 software (Lumenera Corporation, Ottawa, ON, Canada). Samples were considered negative after the entire sediment sample was scanned and no eggs were found. We note that while identification of *Oesophagostomum* eggs was based on a rigorous set of characteristics, this genus cannot easily be distinguished from hookworm infection by eggs alone. However, hookworms have not been found in previous surveys of the gastrointestinal parasites of this primate community [19], [20], suggesting that eggs identified with morphological characteristics of both *Oesophagostomum* and hookworm were almost certainly *Oesophagostomum*.

### Molecular Methods

DNA was extracted from 200 µL of sedimented feces using a ZR Fecal DNA MiniPrep Kit (Zymo Research Corporation, Irvine, CA, USA), following manufacturer protocols. External PCR was performed targeting the ribosomal internal transcribed spacer 2 gene using primers NC1 (5′-ACGTCTGGTTCAGGGTTGTT-3′) and NC2 (5′-TTAGTTTCTTTTCCTCCGCT-3′), which generated products that ranged in size from 280 to 400 bp, suggesting that, as expected, the primer set detected a number of parasitic helminths present in the samples [27], [36]. Subsequently, an internal, semi-nested PCR generating amplicons of predicted size 260 bp was performed using primer NC2 and newly designed *Oesophagostomum*-specific primer, OesophITS2-21 (5′-TGTRACACTGTTTGTCGAAC-3′). Primer OesophITS2-21 was generated by aligning publicly available sequences of the *Oesophagostomum* internal transcribed spacer 2 gene [26], [36]–[39], and GenBank accession numbers HQ283349, HQ844232]. In total, eight species of *Oesophagostomum* were represented in the alignment. Other species of varying relatedness, including other members of the taxa Chabertiidae (*Chabertia ovina*, Accession No. JF680981; *Ternidens deminutus*, Accession No. HM067975), Strongylidae (*Strongylus vulgaris* [40]), and Strongylida (*Necator americanus* [36], and *Ancylostoma duodenale* [41]) were also included. Priming regions were selected to be identical among all species of *Oesophagostomum* but divergent from the other genera. Primer ITS2-21 was highly specific as confirmed by sequencing, since all PCR products matched *Oesophagostomum* despite the fact that a number of other parasites (including *Strongyloides*, *Necator* and *Trichuris*), were identified in the same samples during microscopic examination.

External PCR was performed in 25 µL volumes using the FailSafe System (Epicentre Biotenchnologies, Madison, WI, USA) with reactions containing 1× FailSafe PCR PreMix with Buffer C, 1 Unit of FailSafe Enyme Mix, 2.5 picomoles of each primer (NC1 and NC2), and 1 µL of template. Reactions were cycled in a Bio-Rad CFX96 platform (Bio-Rad Laboratories, Hercules, CA, USA) with the following temperature profile: 94°C for 1 min; 45 cycles of 94°C for 15 sec, 50°C for 30 sec, 72°C for 90 sec; and a final extension at 72°C for 10 min. Internal PCR was performed in 25 µL volumes using the DyNAzyme DNA Polymerase Kit (Thermo Scientific, Asheville, NC, USA) with reactions containing 0.5 Units of DyNAzyme I DNA Polymerase, 1× Buffer containing 1.5 mM MgCl_2_, 2.5 picomoles of each primer (OesophITS2-21 and NC2), and 1 µL of template. Reactions were cycled with the following temperature profile: 95°C for 1 min; 45 cycles of 95°C for 15 sec, 55°C for 30 sec, 70°C for 90 sec; and a final extension at 70°C for 5 min. Amplicons were electrophoresed on 1% agarose gels stained with ethidium bromide, and purified from gels using the Zymoclean Gel DNA Recovery Kit (Zymo Research Corporation, Irvine, CA, USA) according to the manufacturer's instructions.

Products were Sanger sequenced in both directions using primers OesophITS2-21 and NC2 on ABI 3730xl DNA Analyzers (Applied Biosystems, Grand Island, NY, USA) at the University of Wisconsin-Madison Biotechnology Center DNA Sequencing Facility. Sequences were hand-edited and assembled using Sequencher v4.9 (Gene Codes Corporation, Ann Arbor, MI, USA) and all ambiguous bases were resolved by repeat PCR and re-sequencing, as described above. All new sequences were deposited in GenBank, under Accession Numbers KF250585 - KF250660.

### Phylogenetic Analyses

Sequences were aligned using the computer program ClustalX [42] with minor manual adjustment. Published reference sequences were included to identify putative species (AF136575, Y11733, AF136576) and as outgroups (HQ844232, Y11738, Y11735, Y10790, AJ006149), and were trimmed to the length of the newly generated sequences using Mesquite v.2.75 [43]. Trimmed sequences yielded the same tree topology as did untrimmed sequences (by neighbor-joining method; results not shown), suggesting that the amplified region was sufficient for taxonomic discrimination. Phylogenetic trees were reconstructed using maximum likelihood in MEGA v.5.05 [44] and the Hasegawa-Kishino-Yano substitution model [45]. Phylogenetic support was assessed using 1,000 bootstrap replicates. To estimate *Oesophagostomum* genetic diversity, percent nucleotide-level sequence identity among sequences was calculated as the uncorrected pairwise proportion of nucleotide differences (p-distance) in MEGA v5.05 [44].

### Statistical Analysis

Diagnostic performance of microscopy *versus* PCR was estimated by calculating sensitivity (*i.e.*, true positive rate) and specificity (*i.e.*, true negative rate) using MedCalc v.12.5.0 (MedCalc Software, Ostend, Belgium). Prevalence of infection was calculated as the number of samples found to be positive for *Oesophagostomum* divided by the total number of samples collected, with 95% confidence intervals calculated using the modified Wald method [46]. To determine whether prevalence differed among primate host species, a chi-square test was conducted in Quantitative Parasitology v3.0 [47]. To explore variation in prevalence among hosts while controlling for their phylogenetic non-independence, a phylogenetic least squared regression (PGLS) was conducted in R [48] using the ape [49] and caper [50] libraries. Prevalence of *Oesophagostomum* was included as the dependent variable, and various primate life history traits were independent variables: terrestriality (predominantly terrestrial *versus* predominantly arboreal), maximum home range [51]–[56], maximum group size [51], [53], [55], [57]–[60], percentage time spent in polyspecific associations [61], [62], average female body mass, and average daily travel distance (the latter was log transformed since the relationship was close to exponential) [55], [56], [62]–[64]. Humans were omitted from the PGLS analysis because many of these traits vary widely among human populations, making accurate estimations problematic.

To determine the degree to which each *Oesophagostomum* lineage (*i.e.*, taxonomic unit) identified by DNA sequencing was host restricted, we calculated the phylogenetic dispersion of infected hosts using the net relatedness index (NRI) in R [48] using the ape [49] and picante libraries. Mean pairwise distance (MPD) was weighted by the ratio of occurrence of each *Oesophagostomum* within each lineage, and compared to null expectation in 1000 randomly assembled communities. Results are reported as standard effects sizes, with values close to 1 indicating phylogenetic evenness (*i.e.*, *Oesophagostomum* lineages infect a greater diversity of hosts than would be expected by chance), while values <0.05 indicate phlylogenetic clustering (*i.e.*, *Oesophagostomum* lineages are host-specific).

## Results

**Figure 2:**
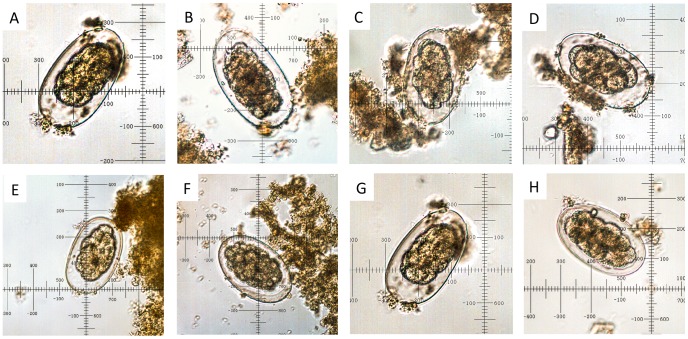
Microscopic images of representative *Oesophagostomum* sp. eggs found in the feces of infected primate hosts. Images were captured at 40× objective magnification from thin smears of sedimented feces. A = blue monkey, B = black and white colobus, C = chimpanzee, D = l'hoest monkey, E = grey-cheeked mangabey, F = olive baboon, G = red-tailed guenon, H = human. All eggs were between 65 and 84 µm long and between 35 and 55 µm wide.

A total of 318 fecal samples from primates, including humans, were collected (Table 1). Of these, 112 were positive for *Oesophagostomum* by microscopy, for a community-wide prevalence of infection of 35.2% (Table 1). All eggs identified by microscopy were similar in internal and external morphology in samples from all primate species (Figure 2). Eggs were 65–80 by 35–50 µm in size, which is consistent with previous results from this community [19], [20] (Figure 2).

**Table 1:** Prevalence of *Oesophagostomum* spp. in nine primate host species (including humans) in and near Kibale National Park, Uganda, based on microscopy and PCR.

		Number positive		Prevalence (95% CI)	
Species	N	Microscopy	PCR	Microscopy	PCR
BM (Blue monkey)	33	10	24	30.3 (17–47)	72.7 (56–85)
BW (Black and white colobus)	37	8	21	21.6 (11–37)	56.8 (41–71)
CH (Chimpanzee)	30	18	30	60.0 (42–75)	100 (86–100)
GM (Gray-cheeked mangabey)	42	17	39	40.5 (27–56)	92.9 (80–98)
HU (Human)	36	3	9	8.3 (2–23)	25.0 (14–41)
LM (L'hoest monkey)	8	6	8	75.0 (40–94)	100 (63–100)
OB (Olive baboon)	27	18	27	66.7 (48–81)	100 (85–100)
RC (Red colobus)	64	11	26	17.2 (10–28)	40.6 (29–53)
RT (Red-tailed guenon)	41	21	38	51.2 (36–66)	92.7 (80–98)
TOTAL	318	112	222	35.2 (30–40)	69.8 (65–75)

PCR generated single, clear amplicons of expected size (260 bp) in 222 samples, indicating positive detection of *Oesophagostomum* DNA, for an overall prevalence of 69.8%. No amplicons were present in remaining samples. Resulting DNA sequences overlapped 100% with published sequences and contained no insertions or deletions, making alignment unequivocal.

When PCR results were compared to microscopy, the overall sensitivity of PCR was 100% (95% CI 96.8%–100.0%), but specificity was only 47.5% (95% CI 40.5%–54.7%). Thus, PCR did not classify any microscopy-positive samples as negative but identified 109 microscopy-negative samples as positive.

Prevalence of *Oesophagostomum* infection (as determined by both microscopy and PCR) varied significantly among host species (microscopy: chi-square = 54.31, df = 8, *P*<0.0001; PCR: chi-square = 112.2, df = 8, *P*<0.0001). Both microscopy and PCR identified humans as having the lowest prevalence of infection (8.3% and 25.0%, respectively), followed by red colobus (17.2% and 40.6%, respectively). Chimpanzees, l'hoest monkeys, and olive baboons had the highest prevalence by both methods, with 100% prevalence by PCR in all three species (although sample sizes were low in some cases; Table 1).

PGLS indicated that terrestriality, maximum home range, maximum group size, percent time spent in polyspecific associations, and average female body mass were not significant univariate predictors of *Oesophagostomum* prevalence (all *P*>0.05 from PGLS with lambda = ML; Table 2). However, log daily travel explained nearly 55% of the variation in prevalence among host species (*P*<0.05, R^2^ = 0.546, from PGLS with the ML estimate of lambda = 0). In a multivariate model, both group size and log daily travel were significant predictors of prevalence, with group size showing a negative relationship and log daily travel a positive relationship (Table 2). This two-predictor model including group size and daily travel explained over 75% of the variation in *Oesophagostomum* prevalence among species (model *P*<0.01, R^2^ = 0.7701; Table 3).

**Table 2:** Phylogenetic generalized least-squared multiple regression models of the relationship between *Oesophagostomum* prevalence and univariate life-history variables of diurnal primates (excluding humans) within the Kibale community.

Univariate Model	λ	Slope	F	*P*	Adjusted r^2^
Terrestriality	0.97	0.20495	2.93	0.130	0.2159
Home range	1.00	0.00004	1.07	0.400	0.0101
Group size	1.00	−0.00023	0.05	0.950	−0.1567
Polyspecific association	1.00	−0.19713	1.16	0.375	0.0225
Body mass	1.00	0.00400	0.61	0.578	−0.0590
Log daily travel	0.00	0.18500	9.43	0.014	0.5464

**Table 3:** Phylogenetic generalized least-squared multiple regression models of the relationship between *Oesophagostomum* prevalence and life-history variables of diurnal primates (excluding humans) within the Kibale community.

Multivariate Model	λ	Slope	t	*P* trait	F	*P* overall	Adjusted r^2^
y-intercept	0.00	−1.93390	−2.65	0.045	9.95	0.015	0.7189
Home range +		0.00012	−2.16	0.083			
Log daily travel		0.00039	3.70	0.014			
y-intercept	0.00	−0.89378	−2.58	0.049	12.73	0.009	0.7701
Group size +		−0.00180	−2.61	0.047			
Log daily travel		0.24894	5.04	0.004			
y-intercept	0.00	−1.48140	−1.97	0.120	8.39	0.032	0.7599
Home range +		−0.00006	−0.88	0.425			
Group size +		−0.00127	−1.36	0.245			
Log daily travel		0.33085	3.14	0.035			

**Figure 3:**
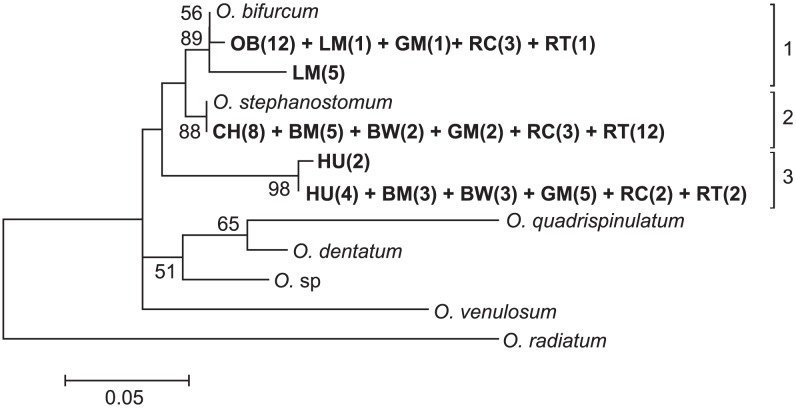
Phylogenetic analysis of *Oesophagostomum* based on ITS2 rDNA (260 bp) sequences. Nucleotide sequences were aligned using Clustal X software [42]. Phylogenetic relationships were inferred in MEGA5 [44], using the maximum likelihood method with a Hasegawa-Kishino-Yano model of nucleotide substitution [45]. The best-scoring maximum-likelihood tree is shown here (−lnL = 656.5). Bootstrap values (%) greater than 50% are shown. Taxon names of sequences generated in this study are in bold and correspond to the host species followed by the number of infected individuals in parentheses (BM = blue monkey, BW = black and white colobus, CH = chimpanzee, LM = l'hoest monkey, GM = grey-cheeked mangabey, OB = olive baboon, RC = red colobus, RT = red-tailed guenon, and HU = human). Reference sequences correspond to Genbank accession numbers AF136575 and Y11733 for *O. bifurcum*, AF136576 for *O. stephanostomum*, HQ844232 for *O. sp*, Y11738 for *O. quadrispinulatum*, Y11735 for *O. dentatum*, Y10790 for *O. venulosum*, and AJ006149 for *O. radiatum*. Scale bar indicates nucleotide substitutions per site.

From 222 positive samples, 76 were randomly selected for sequencing to represent as even a number of positive samples per host species as possible. All 76 sequences most closely matched published *Oesophagostomum* ITS-2 DNA sequences using the BLASTn tool on the National Centre for Biotechnology Information website. Phylogenetic analysis resolved these sequences into three clades (Figure 3). Clade 1 contained all 12 sequences from olive baboons, one sequence from l'hoest monkeys, one sequence from grey-cheeked mangabeys, three sequences from red colobus and one sequence from red-tailed guenons. These sequences were identical to published reference sequences for *O. bifurcum* [26], [36]. Five additional sequences from l'hoest monkeys sorted into clade 1 and were 97.1% similar to this same *O. bifurcum* reference sequence. Clade 2 contained all eight sequences from chimpanzees, five sequences from blue monkeys, two sequences from black and white colobus, two sequences from grey-cheeked mangabeys, three sequences from red colobus, and twelve sequences from red-tailed guenons. All sequences in clade 2 were identical to an *O. stephanostomum* reference sequence [26]. Clade 3 was composed of two nearly identical branches (99.4% identity) that contained all six sequences from humans, as well as sequences from three blue monkeys, three black and white colobus, five grey-cheeked mangabeys, two red colobus, and two red-tailed guenons. These sequences were 92.4–93.0% and 93.0–93.6% similar to *O. bifurcum*, and *O. stephanostomum*, respectively, but were not identical to any published reference sequence.

Host species were phylogenetically clustered within *O. bifurcum* clade 1 (NRI = −1.76, *P*<0.05). Clade 2 (*O. stephanostomum*) did not vary significantly from the null expectation of no host clustering, NRI = 0.86, *P* = 0.75). Clade 3 was marginally phylogenetically over-dispersed with respect to distribution of host species (NRI = 1.24, *P* = 0.04).

## Discussion

Here we evaluate the prevalence of *Oesophagostomum* infection in wild primates and humans in Western Uganda using both microscopy and PCR. Our results clearly show that prevalence varied significantly among host species. Humans had the lowest prevalence of infection likely because of avoidance behaviors such as sanitation practices [65], [66] and because of the common use of antihelminthics in the region. Red colobus and black and white colobus also had comparatively low prevalence of infection, as found in previous studies [16], [20], [67]. This observation may reflect colobine gastrointestinal physiology, which is characterized by folivory and foregut fermentation [68], and the associated regular ingestion of plant secondary compounds that may suppress infection by pathogenic organisms [69]. Conversely, the high prevalence of infection in chimpanzees, olive baboons, and l'hoest monkeys may reflect reduced physiological barriers to infection or increased susceptibility. To explain this interspecific variation in prevalence, we examined correlations between life history variables and prevalence among host species. We found that two variables, daily travel distance and group size, explain over 75% of the variance in *Oesophagostomum* prevalence among host species. Surprisingly, body mass, the strongest predictor of helminth species richness elsewhere, was not significant here [67].

Previous studies have concluded that group-living animals with small home ranges are likely to suffer high intensities of infection due to frequent environmental re-exposure [70]–[72]. Our results indicate the opposite in the case of *Oesophagostomum*: smaller primate groups with large daily travel distances had higher prevalence. Animals with larger day ranges may encounter greater habitat variation [73], which may increase exposure to *Oesophagostomum* from environmental sources. In addition, previous research has implicated terrestriality as an important factor affecting the prevalence of trematode parasites in primates [74]. In our study, the three host species with highest *Oesophagostomum* prevalence (chimpanzees, olive baboons and l'hoest monkeys) were also the only three predominantly terrestrial species. Although this trend was not statistically significant, it is possible that terrestrial primates contact soil more frequently, and thus the infective stages of STHs.

Although group size was not a significant predictor of prevalence in univariate analyses, our multivariate analysis found smaller groups with large daily travel distances to be at greatest risk of infection. This finding contrasts with previous studies showing that increased intragroup contact increases exposure [71], [75]. In Kibale, positive associations between group size and parasite richness have been documented for protozoan parasites in mangabeys [76]. However parasite richness is not necessarily associated with prevalence. Small primate groups might maintain high intra-group infection rates for certain parasites if transmission within the group is frequent, thus maintaining high prevalence (as seen here) without correspondingly high parasite richness.

Our study detected substantial cryptic phylogenetic diversity in *Oesophagostomum* infecting Ugandan primates. Currently, the principal human *Oesophagostomum* species is considered to be *O. bifurcum* [5], while other great apes harbor *O. stephanostomum* [6], [12], [18], [77]. Recently, however, chimpanzees inhabiting a northern sector of Kibale were identified as positive for *O. bifurcum*, making this the first discovery of *O. bifurcum* in non-human apes. The same study identified chimpanzees also infected or co-infected with *O. stephanostomum* [22]. In our phylogenetic analysis, we identified both *O. bifurcum* and *O. stephanostomum* in the Kibale primate community. However, we found only *O. stephanostomum* in chimpanzees, although the possibility of undetected *O. bifurcum* infections cannot be ruled out.

In addition, we identified a third *Oesophagostomum* lineage that did not cluster with any published sequence and thus may represent a previously uncharacterized taxon. It is possible that this new taxon has remained undetected in previous molecular investigations. We examined the OB primer that has been used previously to identify *O. bifurcum* [36] and conclude that it would probably not amplify our newly identified taxon due to mismatched bases at both the 5′ and 3′ ends of the primer. It is therefore possible that the new taxon we identified exists elsewhere (e.g. in Togo and Ghana) but has been not been detected or differentiated from other members of the genus. However, we caution that these inferences are based on a short region of a single gene, and that sequencing additional genes as well as morphological characterization of L3 larvae and adults will be necessary to confirm these findings. Nonetheless, our results suggest a heretofore unappreciated degree of hidden genetic diversity within this well-described genus of parasites that are known to infect humans.

Interestingly, all *Oesophagostomum* sequences recovered from humans clustered with the previously undescribed third taxon, and not with published *O. bifurcum* sequence from humans elsewhere in Africa (clade 1) [36]. In Ghana, geographic separation between humans and non-human primates infected with *Oesophagostomum*, despite apparently conducive environments for zoonotic transmission, motivated efforts to determine the host range of the parasite using molecular methods [28]. Genome-wide analyses (amplified fragment length polymorphism, random amplification of polymorphic DNA) suggested that *O. bifurcum* clusters into distinct groups by host species, thus suggesting that zoonotic transmission is uncommon [4], [17]. By contrast, in our study area, no such geographic separation exists between humans and non-human primates. In this setting, we found that both humans and non-human primates were infected with the novel *Oesophagostomum* clade 3, which is phylogenetically over-dispersed compared to the other *Oesophagostomum* clades. While our conclusions await verification from more detailed examination of the *Oesophagostomum* genome, our results nevertheless suggest that this novel clade may be broadly transmissible among species of distantly related primate hosts, including humans. The Kibale ecosystem is known for its high degree of spatio-temporal overlap between humans and non-human primates and its ensuing high rates of transmission of diverse pathogens across primate species [78]–[81]. Our results provide further evidence for cross-species pathogen transmission between wild primates and humans in this region.

Our paired analyses applying both microscopy and PCR to the same samples indicate that traditional methods based on microscopy may significantly underestimate prevalence. Concentration methods followed by microscopic visualisation of eggs in thin smears are considered definitive diagnostic methods for soil-transmitted helminth (STH) infections [82], [83]. Previous studies that have used fecal sedimentation and microscopy have reported *Oesophagostomum* infection prevalence estimates between approximately 3% and 10% in wild Ugandan primates [19], [20]. These values are considerably lower than what we report here using molecular methods; however, our results parallel other studies that have estimated prevalence using molecular methods [16], [22]. Not surprisingly, we find that PCR is more sensitive than microscopy, perhaps because it can detect *Oesophagostomum* infection even when eggs are not present. For example, tissues or secretions shed by adult worms into the intestinal lumen would be detected by PCR, as would eggs that have hatched into L1 larva prior to fixation during the sedimentation procedure.

To our knowledge, ours is the first study in several decades to report human *Oesophagostomum* infection in Uganda, a country that is over 3,000 km from known foci of infection in West Africa [5]. Given that the prevalence of *Oesophagostomum* was 25% in our sample of people, we suspect that this parasitic infection occurs more commonly across Sub-Saharan Africa than previously thought and may be causing infections that are untreated or misdiagnosed. Our finding of a previously genetically uncharacterized lineage of *Oesophagostomum* that may be transmitted among primate species underscores that the diversity (genetic and otherwise) of this parasite genus may be under-sampled in Africa. Further ecological studies of *Oesophagostomum* in Uganda and elsewhere are needed to quantify the degree of enzootic versus zoonotic transmission. Regardless of the outcome of such research, our results suggest that *Oesophagsotomum* should be considered a pathogen of concern beyond its accepted foci of infection in Togo and Ghana, and perhaps across all of equatorial Africa.
